# The comparison of the 1972 Hodkinson’s Abbreviated Mental Test Score (AMTS) and its variants in screening for cognitive impairment

**DOI:** 10.1007/s40520-018-1009-7

**Published:** 2018-07-30

**Authors:** Karolina Piotrowicz, Wojciech Romanik, Anna Skalska, Barbara Gryglewska, Katarzyna Szczerbińska, Jarosław Derejczyk, Roger M. Krzyżewski, Tomasz Grodzicki, Jerzy Gąsowski

**Affiliations:** 1 Department of Internal Medicine and Gerontology, Faculty of Medicine, Jagiellonian University Medical College, 10, Śniadeckich St., 31-351 Kraków, Poland; 2 II Department of Anesthesiology and Intensive Care, Central Teaching Hospital, Medical University of Warsaw, Warsaw, Poland; 3 Faculty of Medicine, Jagiellonian University Medical College, Kraków, Poland; 4 John Paul II Geriatric Hospital, Katowice, Poland; 5 Department of Neursurgery and Neurotraumatology, Faculty of Medicine, Jagiellonian University Medical College, Kraków, Poland

**Keywords:** AMTS, MMSE, Cognitive impairment, Validation, Mental test

## Abstract

**Background:**

Since its introduction by Hodkinson in 1972, Abbreviated Mental Test Score (AMTS) and its English and other language versions have been widely used in research and clinical practice alike. However, whether the various versions of AMTS yield equivalent information has never been tested.

**Methods:**

We performed cross-sectional assessment of inpatients aged 65+ years with seven AMTS versions and the Mini-Mental State Examination (MMSE) after correction for age and education (MMSEc). We used the MMSEc cut-off score of < 24 as comparator and fitted linear regression models from which we obtained the receiver operating characteristics, and further compared the *c*-statistics obtained for each version of AMTS. We used Spearman’s correlation to check the relation between different AMTS versions.

**Results:**

The mean (SD) age of 72 (52.8% women) patients was 76.2 (7.6) years. The average time spent on education was 11.3 (3.5) years. The AMTS score across versions varied between 7.4 (2.0) and 8.2 (1.7). The MMSE averaged 24.1 (4.6) and the MMSEc averaged 25.2 (4.1). We found that the *c*-statistic across AMTS versions with dichotomised MMSEc as comparator ranged from 0.83 to 0.85 and did not significantly differ from the *c*-statistic of 0.87 for original AMTS (all *p* > 0.16). We found AMTS versions to be significantly correlated (all *r* between 0.83 and 0.99, all *p* < 0.0001).

**Conclusions:**

We found AMTS to be a reliable and useful tool in the screening for possible cognitive impairment. This seems to be true irrespective of whether we use the original test or any of its studied modifications.

## Background

The Abbreviated Mental Test Score (AMTS) was first introduced in 1972 by Hodkinson for the assessment of mental impairment in old age [1]. AMTS is a 10-item screening questionnaire that included in the original version questions about: subject’s age, date of birth, the current time and year, the name of hospital, the name of the present British monarch, the year of the beginning of the First World War; and a recall of a previously given address, counting backwards by 1, from 20 to 1, and recognition of two persons (e.g., doctor, nurse, etc.) [2].

Based on the search of literature, we found several English language modifications of AMTS [3–6] (summarized in Table 1). We also found a Polish version of AMTS [7, 8] which has been widely used both in clinical practice [8] and research [9] (Table 1).

**Table 1:** The components of the AMTS tests used in the study

Original test by Hodkinson and modifications	Q1	Q2	Q3	Q4	Q5	Q6	Q7	Q8	Q9	Q10
Hodkinson [1, 2]	Age	Time (to the nearest hour)	Address for recall at the end of test—this should be repeated by the patient to ensure it has been heard correctly: 42 West Street	Year	Name of hospital	Recognition of two persons (doctor, nurse, etc)	Date of birth	Year of First World War	Name of present monarch	Count backwards 20–1
Polish [7]	–	–	[The repetition of the address scores]	–	Address	Recall of the previously memorised address [this comes as last question in the test]	–	Year of Second World War	Name of current Polish president	–
Hodkinson (WW2) [6]	–	–	–	–	Current location	–	–	Year of Second World War	–	–
Hodkinson (current location) [6]	–	–	–	–	Current location	–	–	–	–	–
RCGP cook and postman [3]	–	–	–	–	Address	Recognition of two persons (postman, cook)	–	–	–	–
RCGP the Pope and the Queen [3]	–	–	–	–	Address	Recognition of two persons (Pope, Queen)	–	–	–	–
RCGP (the Pope and Lech Walesa)	–	–	–	–	Address	Recognition of two persons (Pope, Lech Walesa)	–	–	–	–

The ellipses denote lack modification when compared to original test by Hodkinson. Current location meant name of hospital or town

*WW2* Second World War, *RCGP* the Royal College of General Practitioners

Several papers described validations of some of national versions of AMTS [10–15], or the validity of the original version as published by Hodkinson in 1972 in detecting persons with definite dementia, according to the clinical criteria prevailing during the execution of particular study [16–19], or suspected dementia (based on the MMSE score) [17, 20–22]. However, to the best of our knowledge the widely circulated versions of AMTS, including the Polish modification, have never been compared between one another in terms of their value in detecting suspected cognitive impairment.

## Methods

The aim of the study was to compare the basic diagnostic properties of the Polish translation [7], the English versions published in the Occasional Paper by the Royal College of General Practitioners (RCGP) [3, 4], and the original questionnaire of the Abbreviated Mental Test Score [1, 2]. We employed the Mini-Mental State Examination (MMSE) after correction for age and years of education (MMSEc), as a reference test for suspicion of cognitive impairment, with the MMSEc cut-off score of 24 points (23 or below) [23]. To adjust the results for age and education we used the correction as proposed by Mungas et al. [24]. The threshold score for the cognitive impairment in AMTS was 7 points (6 or below). The comparison of the questionnaires of the original, the Polish version and the English language derivatives of the original AMTS is presented in Table 1. When testing the ability to recognise persons, in the RCGP version of AMTS we used the pictures of a postman and a cook, and the Pope John Paul II and the Queen Elizabeth II. Additionally, we decided to adapt the RCGP version for the Polish clinical environment, and proposed to use a picture of Lech Wałęsa, the first democratically elected president of Poland since the fall of the East Bloc, instead of a picture of the Queen. The study was performed with the approval of the Ethics Committee of the Jagiellonian University, Kraków, Poland (No.: 122.6120.191.2016), and all subjects gave their informed consent to take part in it. All versions of AMTS were translated and back-translated, as applicable, between the English and the Polish by separate members of the research team (KP, JG). We used the validated Polish version of the MMSE [25]. The eligible subjects had to be aged 65 years or more, and they had to be free from an acute disease. The included patients were the inpatients of Dept. of Internal Medicine and Geriatrics, University Hospital, Kraków, Poland or the patients under assessment for planned surgical procedure at the Central Teaching Hospital, Medical University of Warsaw, Warsaw, Poland. The acutely admitted patients were assessed after stabilization of their medical condition.

The data management and analysis were performed with SAS 9.2 (SAS Institute Inc. Cary, NC, USA). Means were compared with Student’s *t* test, proportions with Chi-square test. We employed Spearman’s correlation to check for the associations between the respective AMTS versions and the original AMTS as published by Hodkinson in 1972. Based on the logistic regression models with the aforementioned classification based on MMSEc as dependent variable, we assessed the receiver operating characteristics (ROC) for seven versions of AMTS (Table 1). We used the *c*-statistics as estimates of area under the curve (AUC). We compared the AUC for the original AMTS and the AUC for the Polish version of AMTS with the AUC of every other AMTS studied. The comparisons were based on Wald’s standard errors and 95% confidence intervals. All significance levels were two-tailed with *p* < 0.05 indicating statistical significance.

## Results

We collected data of 72 consecutive patients (52.8% women) aged 65 years and older with mean (SD) age of 76.2 (7.6) years, and 11.3 (3.5) years of formal education. The MMSE averaged 24.1 (4.6) and the MMSEc averaged 25.2 (4.1). Based on the unadjusted MMSE results, 38.7% of the subjects had the suspicion of cognitive impairment. After the correction for age and years of education [24] the percentage of subjects with the MMSE < 24 was 26.0%. The AMTS score across versions varied between 7.4 (2.0) and 8.2 (1.7). Based on the AMTS, the suspicion of cognitive impairment ranged from 12.3 to 21.9%. The inter-version AMTS correlations ranged from 0.83 to 0.99 (all *p* < 0.0001). The concordances (AUC) between studied AMTS versions and the classification of patients based on MMSEc ranged from 0.83 to 0.87 and did not significantly differ (all *p* > 0.10, Table 2).

**Table 2:** The AUC statistics based on the ROC analysis between the MMSEc-based suspicion of cognitive impairment (corrected for age and years of education) and the tested versions of the AMTS

ROC model	Mean (SD) AMTS score	Area	SE	95% Wald CI	*p* value vs. Hodkinson	*p* value vs. Polish
Hodkinson [1, 2]	7.4 (2.2)	0.87	0.04	0.79–0.95	–	0.44
Polish [7]	8.2 (1.7)	0.85	0.05	0.75–0.95	0.44	–
Hodkinson (WW2) [6]	8.0 (1.8)	0.83	0.05	0.73–0.93	0.18	0.10
Hodkinson (current location) [6]	7.7 (1.8)	0.84	0.05	0.74–0.94	0.20	0.78
RCGP cook and postman [3]	7.7 (1.8)	0.83	0.05	0.73–0.93	0.16	0.59
RCGP the Pope and the Queen [3]	7.6 (1.9)	0.83	0.05	0.73–0.93	0.17	0.50
RCGP (the Pope and Lech Wałęsa)	8.1 (1.8)	0.85	0.05	0.75–0.95	0.49	0.62

*p* values are for comparisons of respective AUC with the AUC based on the original Hodkinson or the Polish versions, respectively

*AUC* area under the curve, *ROC* receiver operating characteristic, *SE* standard error, *CI* confidence interval, *WW2* Second World War, *RCGP* the Royal College of General Practitioners

## Discussion

In this cross-sectional study of 72 older subjects, we found that AMTS and its derivative versions had good (*c*-statistic from 0.83 to 0.87) concordance with MMSE after correction according to Mungas et al. [24] in determining a suspicion of cognitive impairment. This does not significantly differ across various English language versions of AMTS currently in use, and the Polish modification of the test. This is reflected by a significant correlation between scores obtained from different versions of AMTS.

The practical universal use of AMTS poses several problems. First, changes at the societal level, education, and the sheer weight of more recent historical events that draw attention of the societies and the individuals alike may raise suspicion that questions pertaining to such entities as the beginning of great wars (WW1 vs. WW2) or the rule of British Monarchs, may have different weight currently than they had in the past [8, 14]. The literature abounds in the modifications of the original inventory [3–6, 8, 14, 15, 26]. In addition, many versions other than those in English language have been created, usually at the time of systemic transformation in Central and Eastern Europe, the Gulf Region, or Far East. Some local versions may not have been based on original work by Hodkinson of 1972, instead, relying on more easily accessible, more recent English language modifications of the original [8].

The English and non-English language versions of AMTS vary on items testing patient’s orientation and memory, and visuospatial abilities [3, 4, 6, 7, 11, 13–15, 19].

Several national validations have been published [11, 13–15, 19]. The validation of the Iranian version substitutes the date of WW1 with the Iraqi–Iranian war and the name of the Queen with the name of the then current Iranian leader [14]. In the validation study conducted in Hong Kong, the authors replaced the date of WW1 with the date of mid-Autumn festival and the name of the Queen with the name of current Governor of the Chinese leader [15], an Italian validation substituted the name of the Queen with the name of the Italian president [19]. We found that similar cultural and historical substitutions made to the original test across the modified AMTS versions we studied did not markedly interfere with the informative value of the tests (Table 2). In this respect, our results lend formal support to the comparisons of the AMTS value in detecting cognitive impairment as published in review papers [17].

Our study should be considered in the context of its possible limitations. First, the patients included were not randomly drawn, instead they were consecutive inpatients drawn from two centers in Poland and did not include any persons for whom English would have been a mother tongue. Thus, the tests were administered in Polish. However, the simple and unequivocal nature of the questions, confirmed in the translation/backtranslation process, left little room for bias. Another limitation may be attributed to the fact the variants of the test were administered as added on top of the core of the inventory (see Table 1). However, this was done to exclude interpretational problems which might arise due to a learning phenomenon potentially associated with repeated administration of the core inventory [27]. As a standard we used MMSE (validated Polish version purchased by WR and JG, http://www.en.practest.com.pl/node/28991) which only approximates mental function. However, the test has been extensively used for research and clinical purposes worldwide. To minimise the probability of type II error, we performed the sample size assessment which indicated that our study had approximately 80% power to detect the difference between ROC curves of 0.2 (0.95 vs. 0.75) with 5% significance, with the assumption that under the null hypothesis the ROCs under comparison would equal 0.85.

In conclusion, in line with some of the previous studies [10, 12, 17, 22, 28, 29], we found AMTS to be a reliable and useful tool in the screening for possible cognitive impairment with Mungas-adjusted MMSE of < 24 as comparator. This seems to be true irrespective of whether we use the original test or any of its studied modifications. Despite the fact that the study was performed on Polish population, the implications extend well beyond a single country. Thus far, to the best of our knowledge, no study formally addressed the issue of whether different variants of AMTS currently in use would perform equally against a standard, broadly used measure such as MMSE. We show that indeed they do, and that the ROCs revolve about the value of 0.85. Previously, we showed that subclinical changes in the AMTS correlate with poor compliance with prescribed antihypertensive regimen and worse blood pressure control in a randomly drawn cohort of 1988 community living older hypertensive patients [9]. Our current data give support for the continued use of this important brief assessment tool in daily practice, especially outside of the specialized geriatric setting.

## Key points

- AMTS is an easy to perform tool for screening assessment of cognition in older subjects.
- Many modifications of the test exist, however whether their screening value is comparable has never been tested.
- We found that irrespective of modifications of questions in AMTS, its screening performance is satisfactory.
- We advocate use of AMTS and its variants in the quick cognitive screening of older patients.
